# Malaria in North-East India: Importance and Implications in the Era of Elimination

**DOI:** 10.3390/microorganisms7120673

**Published:** 2019-12-10

**Authors:** Devojit Kumar Sarma, Pradumnya Kishore Mohapatra, Dibya Ranjan Bhattacharyya, Savitha Chellappan, Balasubramani Karuppusamy, Keshab Barman, Nachimuthu Senthil Kumar, Aditya Prasad Dash, Anil Prakash, Praveen Balabaskaran Nina

**Affiliations:** 1 ICMR-National Institute for Research in Environmental Health, Bhopal, Madhya Pradesh 462030, India; dkbiotek@gmail.com; 2 ICMR-RMRC, NE Region, Dibrugarh 786001, Assam, India; pkmdibr@gmail.com (P.K.M.); drbhattacharyya@yahoo.com (D.R.B.); 3 NITM (ICMR), Belagavi 590010, India; savithachellappan@gmail.com; 4 Department of Geography, Central University of Tamil Nadu, Tiruvarur 610005, India; geobalas@cutn.ac.in; 5 State NVBDCP Unit, Directorate of Health Services, Govt. of Assam, Guwahati 781005, Assam, India; sphc.nvbdcp.assam@gmail.com; 6 Department of Biotechnology, Mizoram University, Mizoram 796004, India; nskmzu@gmail.com; 7 Central University of Tamil Nadu, Tiruvarur 610005, India; apdash@gmail.com; 8 Department of Epidemiology and Public Health, Central University of Tamil Nadu, Tiruvarur 610005, India

**Keywords:** North-East India, *P. falciparum*, *P. vivax*, *An. baimaii*, *An. minimus*, artemisinin combination therapy

## Abstract

Worldwide and in India, malaria elimination efforts are being ramped up to eradicate the disease by 2030. Malaria elimination efforts in North-East (NE) India will have a great bearing on the overall efforts to eradicate malaria in the rest of India. The first cases of chloroquine and sulfadoxine-pyrimethamine resistance were reported in NE India, and the source of these drug resistant parasites are most likely from South East Asia (SEA). NE India is the only land route through which the parasites from SEA can enter the Indian mainland. India’s malaria drug policy had to be constantly updated due to the emergence of drug resistant parasites in NE India. Malaria is highly endemic in many parts of NE India, and *Plasmodium falciparum* is responsible for the majority of the cases. Highly efficient primary vectors and emerging secondary vectors complicate malaria elimination efforts in NE India. Many of the high transmission zones in NE India are tribal belts, and are difficult to access. The review details the malaria epidemiology in seven NE Indian states from 2008 to 2018. In addition, the origin and evolution of resistance to major anti-malarials are discussed. Furthermore, the bionomics of primary vectors and emergence of secondary malaria vectors, and possible strategies to prevent and control malaria in NE are outlined.

## 1. Introduction

Despite large-scale global efforts to eradicate malaria in the last few years, it still remains a major public health burden with an estimated 219 million cases and 435,000 deaths worldwide in 2017 [1]. Even though, there were an estimated 3 million fewer cases than in 2016 (216 million), no significant progress was made in reducing the global malaria burden during 2015–2017 [1]. It is estimated that India and 15 sub-Saharan African countries were responsible for 80% of the world’s malaria burden [1]. The contribution of India to the world’s malaria cases and deaths is estimated to be 4% [1]. Compared to 2016, India reported a 22% decrease in cases in 2017 [1], and most came from Odisha [2].

Malaria transmission in India occurs pre-dominantly from either *P. falciparum* (*Pf*) or *P. vivax* (*Pv*) [3], however, the prevalence of these species varies greatly across different regions of India. There has been a steady increase in *Pf* cases in the last 4 decades, and as of December 2018, *Pf* was responsible for 48.1% of malaria cases in India [2]. North-East (NE) Indian states along with Odisha, Chattisgarh and Jharkhand contribute most of the *Pf* cases in the country [2]. NE India alone contributes to 12% of India’s *Pf* cases [2].

Assam, Arunachal Pradesh, Meghalaya, Mizoram, Nagaland, Sikkim, Manipur and Tripura make up the eight states of NE India. The state of Sikkim is mostly malaria free, and the few cases reported are thought to be imported cases, and hence will not be discussed. As of 2018, the seven NE states account for 15.2% of the total malaria cases in India [2]. In India, it is estimated that 162.5 million people live in high-transmission areas [4], which includes many parts of the NE. The hilly and forested areas of NE India are mostly inhabited by the tribal population, and they are at the highest risk of malaria [5]. The distribution of *Plasmodium* species varies among NE states. *Pf* is the predominant species in Assam, Mizoram, Tripura and Meghalaya, while *Pv* is dominant in Nagaland, Arunachal Pradesh and Manipur. Apart from *Pf* and *Pv,* the other human malaria parasites, *P. ovale* and *P. malariae* have also been recorded from Assam and Arunachal Pradesh [6,7]. The hot and humid climate aided by the numerous hill streams and its tributaries in these tribal areas support perennial mosquito breeding. The primary malaria vectors in NE India are *An. minimus* and *An. baimaii*. *An. minimus* is part of the Minimus Complex that also includes *An. harrisoni* and An. *yaeyamaensis*. In NE India, only *An. minimus* has been reported so far [8]. *An. baimaii* is part of the *An. dirus* Complex that includes 7 other sibling species. *An. baimaii* is the most prevalent sibling species found in NE along with a focal presence of another sibling species, *An. dirus* X [9]. Apart from these 2 primary vectors, other Anophelines such as *An. annularis, An. phillipinensis/nivipes* and *An. culicifacies* are considered to be secondary vectors of malaria in the region [10]. Due to widespread resistance against both chloroquine (CQ) and sulfadoxine-pyrimethamine (SP), artemisinin-lumefantrine (AL) was introduced as the first line anti-malarial against *Pf* in NE Indian states in 2013 [11,12]. The salient features pertaining to malaria in NE India are outlined in Table 1.

Despite being a hot bed for malaria transmission, malaria intervention studies in NE India have been very challenging due to inaccessibility, difficult terrains, forested areas, administrative issues in the inter-state and international border areas, frequent flooding, poor roads and inadequate network connectivity [5]. Geography and demography of the region, predominance of *Pf* over *Pv*, widespread and early emergence of drug resistance to almost all anti-malarials, and the presence of highly anthropophillic malaria vectors like *An. baimaii* and *An. minimus* throughout the region makes it different from the rest of India in terms of malaria control and elimination. In early 2016, the Indian Government announced the national framework for malaria elimination (2016–2030) [13,14]. This is in line with the larger Global Technical Strategy of the World Health Organization (WHO) [15] and Asia Pacific leaders to eliminate malaria by 2030 [16]. Successful malaria intervention strategies in the NE are critical for India’s target to eliminate malaria by 2030.

## 2. Geography of North-East (NE) States

**Figure 1:**
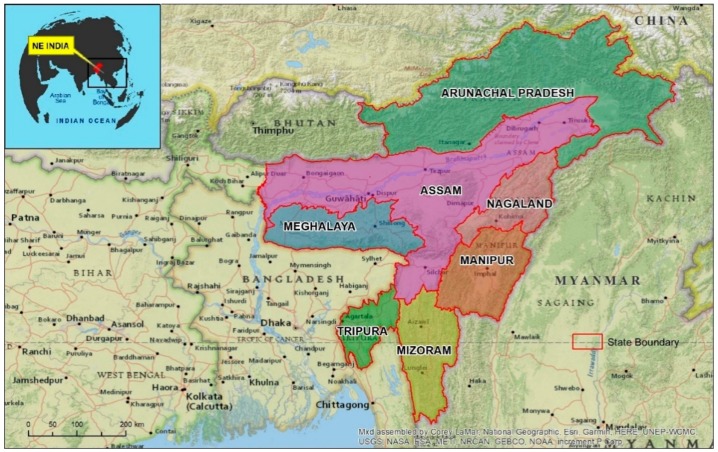
Location map of North-East (NE) India. Red line shows the boundaries of the seven states of NE India.

The NE is largely a mountainous territory with two-thirds of its area occupied by hilly and mountainous terrain with heights ranging from 50 m in Brahmaputra valley to 7000 m above mean sea level in the Himalayan borderland [17]. The states are between 22°00′ to 29°30′ N and 89°40′ to 97°25′ E, and cover an area of 255,128 Km^2^. The NE shares about 4600 Km of international borders with China (on North), Myanmar (on East), Bangladesh (on South) and Bhutan (On West) (Figure 1). The general climate of the NE is tropical monsoon humid climate. The average temperature during winter is 16 °C, and summer is 30 °C. There is a significant climatic contrast between the valleys and the mountainous region. The NE region receives heavy to very heavy rainfall during the South-West Monsoon season (June to September), and rains peak in June. The average annual rainfall is 2000 mm with local variations (1500 to 12,000 mm) [18]. Except for Assam, more than half the geographical area of all NE states is forest. The NE is also one of the two biodiversity hotspots in India [19]. The total population of the region is 45,161,611 (2011 census) and the distribution is not homogeneous. The most densely populated parts of NE region are the plains of Brahmaputra, Barak rivers in Assam, the Imphal plain in Manipur and the western part of Tripura [20].

Assam, located in the central part of NE is considered to be the gateway to NE India as it connects the NE to the Indian mainland by a 22 km strip, called the ‘Chicken’s Neck’, near Siliguri district in West Bengal. Mizoram, Nagaland and Arunachal Pradesh share their international borders with Myanmar (Burma). South East Asia (SEA) is considered to be the hotspot for the generation of drug-resistant parasites. Myanmar is the only land route through which the drug-resistant parasites of SEA can enter India. Thus Mizoram, Nagaland and Arunachal Pradesh, the states bordering Myanmar are strategically important as they can serve as a conduit for the drug-resistant parasites from Myanmar to rest of India.

The NE is inhabited by many tribal groups with unique cultural, social and occupational behavior which have a direct role in malaria transmission. Based on topography and socio-demographic conditions, malaria in the NE region can be classified as tribal malaria, forest malaria, border malaria, and organized sector malaria [21].

## 3. Malaria Epidemiology (2008 to 2018)

**Figure 2:**
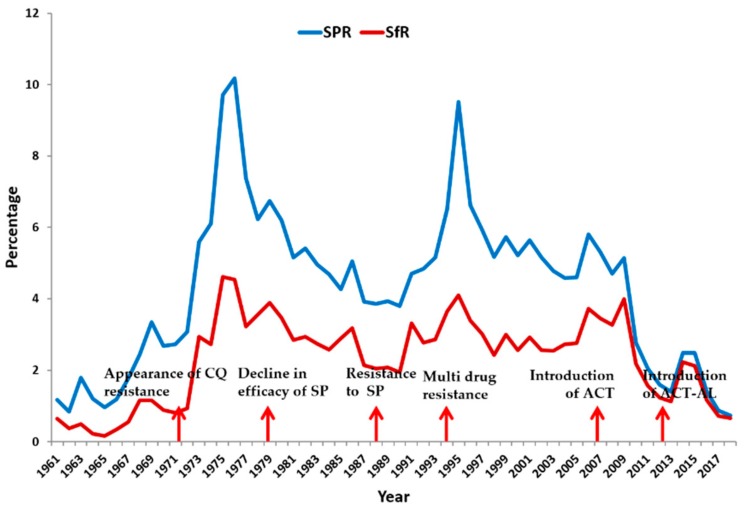
Slide positivity rate (SPR), slide *falciparum* rate (SFR) and emergence of drug resistance in NE states during 1961–2018. Adapted from Mohapatra et al., 2014 [11].

The NE is co-endemic for both *Pf* and *Pv* with a low-to-moderate transmission intensity resulting in intermediate to stable malaria in the whole region [22]. During the eradication era (late 1950s to early 1960s), the NE along with the other states of India witnessed a steady decline in malaria cases. However, the situation reversed and malaria made a comeback in 1966, and peaked in 1976 with 10.2% slide positivity rate (SPR) (Figure 2) (See Box A1 for definition of epidemiological parameters) [21]. The surge in malaria cases was due to the emergence of chloroquine resistance (CQR) in *Pf* and its widespread therapeutic failure all over the NE. Since then, both the SPR and slide *falciparum* rate (SFR) of the NE remained more or less constant until 2008 when CQ was discontinued, and was replaced by artemisinin combination therapy (ACT) as the first line of anti-malarial treatment. Prior to 2008, some major peaks were also observed, and were mostly due to the failure of SP, and emergence of multi-drug resistant parasites in the NE (Figure 2) [11].

**Figure 3:**
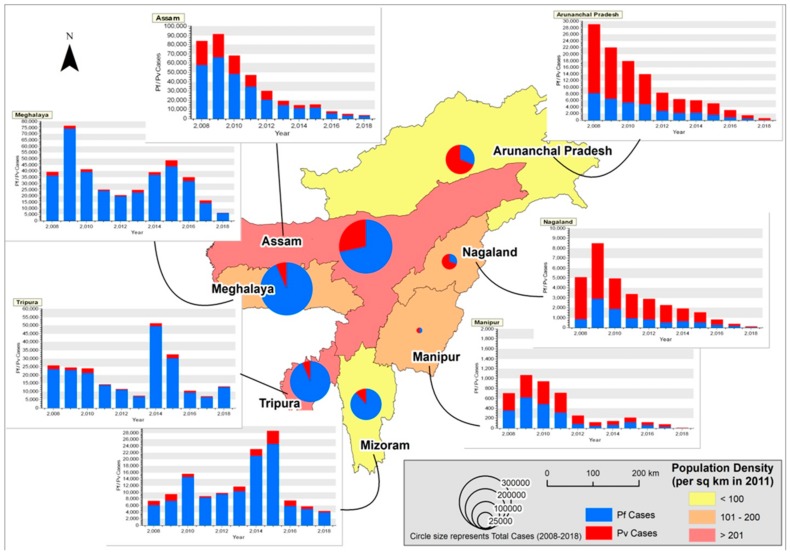
Spatio-temporal distribution of *Pf* and *Pv* cases in NE India during 2008–2018. The background color shows the population density of the states (as per 2011 census). The proportioned pie-chart represents the total number of *Pf* and *Pv* cases reported in the states from 2008 to 2018. (NVBDCP data contains only total malaria cases and *Pf* cases. *Pv* data was obtained by subtracting *Pf* cases from total malaria cases).

Malaria epidemiology data (Table 2) obtained from National Vector Borne Disease Control Program (NVBDCP) website from 2008 to 2018 for 7 NE states are shown spatio-temporally in Figure 3. The State Vector Borne Disease Control Program (SVBDCP) unit will collect information of all malaria cases from block primary health centers (PHCs), sub-centers and private hospitals and will send it to NVBDCP, New Delhi, every month. From 2008 to 2012, Assam reported the maximum number of malaria cases, followed by Meghalaya. The maximum number of cases (91,413, *Pf*-66,557) in the study period was observed in Assam followed by Meghalaya (76,759) in 2009. In Assam, from 2009 onwards, there has been a steady decline in malaria cases, and in 2018, the number of cases recorded were 3816 (*Pf*-2859), below Tripura (13,079) and Meghalaya (6394). In Assam, from a high of 25,815 *Pv* cases in 2008, there has been a continuous decrease, and in 2018, only 957 cases were reported. As of 2018, *Pf* and *Pv* contribute 74.9 and 25.1% of the cases in Assam. Malaria cases in proportion to population is given in Table 3. Among the NE states, Assam has the highest population density (397 persons/sq km). However, based on the cumulative number of cases during the study period (2008–2018), the number of cases per year (0.11%) is comparatively low to the total population. 

Fascinatingly, in Meghalaya, the cases nearly doubled from 39,616 (*Pf*-36,301) in 2008 to 76,759 in 2009 (*Pf*-74,251). From a peak of 76,759 cases in 2009, there was a gradual decrease in cases until 2012 (20,834, *Pf*-19,805). From 2013 to 2015, there was a gradual increase in cases (24,727, *Pf*-22,885 to 48,603, *Pf*-43,828), and this was followed by a steady decline until 2018 (6394 cases, *Pf*-6065). Interestingly, *Pv* cases gradually dropped from 2008 (3315) until 2012 (1029), and was followed by an increase in cases from 2013 (1842) until 2015 (4775), and again was followed by a steep decline until 2018 (329). In Meghalaya, as of 2018, *Pf* and *Pv* contributed 94.8 and 5.2% of the cases. Of the 7 NE states, Meghalaya reported the highest proportion of malaria cases (1.15%/year) to the total population.

In the state of Tripura, there was a steady decline in malaria cases from 2008 (25894, *Pf*-23,588) to 2013 (7396, *Pf*-6998). However, there was a huge malaria epidemic in 2014, and there was a 6.9 fold rise in malaria cases (51240, *Pf*-49,653). Even though there was a gradual decrease from 2015 (32,525, *Pf*-30,074) to 2017 (7051, *Pf*-6571), malaria cases almost doubled in 2018 (13,079, *Pf*-12,600). *Pf* is the leading cause of malaria in Tripura, and contributes to over 90% of the cases. In 2018, *Pv* contributed to just 3.6% of the cases. Tripura’s proportion of malaria cases per year to the total population is 0.55%, and it ranks fourth in malaria endemicity in the 11 year study period.

In Mizoram, the cases increased from 2008 (7361, *Pf*-6172) to 2010 (15,594, *Pf*-14,664), followed by a steep decline in 2011 (8861, *Pf*-8373). From 2012, the cases started increasing again, and a peak of 28,593 cases (*Pf*-24,602) was recorded in 2015. There is a decline from 2016 (7583, *Pf*-5907) to 2018 (4296, *Pf*-3937). In Mizoram, *Pf* predominates, and was responsible for 91.6% of the cases. In Mizoram, the population density is 52 persons per sq km, but the number of cases reported is proportionately very high. Nearly, 1.1 percent of the total population is affected on an average per year, and is the second most malarious state.

The states of Arunachal, Nagaland and Manipur have reported a steady decline in malaria cases from 2009 until 2018, and in 2018, the number of cases reported were 625 (*Pf*-154, *Pv*-471), 113 (*Pf*-24, *Pv*-89) and 12 (*Pf*-3, *Pv*-9) respectively. Fascinatingly, in Arunachal and Nagaland, *Pv* predominates, and in 2018, *Pv* contributed 75.3 and 78.7% of cases respectively. In Arunachal, the average number of cases to population is 0.75%, and is the third highest among all NE states. In Manipur, over the years, both *Pf* and *Pv* have contributed equally to malaria infections. Manipur has the lowest incidence of malaria as the average number of cases to population is just 0.01%.

*Pv* was more in Arunachal Pradesh, Nagaland and Manipur, the states that border Myanmar, while *Pf* predominated in Meghalaya, Tripura, Mizoram and Assam, the states that bordered Bangladesh. A declining trend of malaria was observed in Assam, Arunachal Pradesh, Nagaland and Manipur, the states with higher proportion of *Pv* cases. A cyclical trend was observed in Meghalaya, Tripura and Mizoram dominated by *Pf*.

## 4. Drug Resistance

Historically, SEA has been a hotspot for the generation of drug-resistant parasites. In the last 50 years, SEA has been a fertile ground for developing resistance against pyrimethamine, CQ, SP, quinine, mefloquine, and most worryingly, artemisinins [23]. The only land route through which the parasites of SEA can enter India is through Myanmar. The 4 NE states of India: Mizoram, Manipur, Arunachal and Nagaland share their international borders with Myanmar. Most likely, the drug-resistant parasites originating in SEA would have entered the 4 NE states through Myanmar, and subsequently should have spread to the Indian mainland. CQR originated in the late 1950s in SEA [24], and was first reported in Myanmar (Burma) in 1969. In India, CQR was first reported in the Karbi Anglong district of Assam in 1973 [25]. Following the initial reports of CQR, 13 regional teams were set up in 1978 to monitor anti-malarial resistance through in vivo-efficacy trials [26]. From 1978 to 2007, 54 CQ efficacy studies were carried out in the NE involving 2541 patients, and the treatment failure was 35% (893) [26]. Across India, CQ treatment failure was observed most in NE, and is the probable locus from which CQR would have spread to eastern, followed by western, parts of India [26]. In 1982, a national drug policy was implemented to improve the management of malaria cases, and established SP as the treatment in CQ-resistant regions [26]. In 2005, for CQ treatment failures, artesunate plus SP replaced SP alone as the second-line of treatment, and in regions with documented drug resistance, artesunate plus SP was recommended as the first-line of treatment [26]. In 2007, for regions with documented CQ resistance and high-risk districts, artesunate plus SP was selected as the first-line anti-malarial [26]. From 2010 onwards, artesunate plus SP has been the recommended first-line anti-malarial across India [27]. In 2009, nation-wide sentinel sites were set up to monitor anti-malarial drug resistance [28].

The first report of SP resistance was again in Karbi Anglong district of Assam in 1979 [29]. Drug-resistance studies carried out in Assam during 2006 and 2007 identified 12.6% treatment failure in SP, accompanied by resistant mutations in *dhfr* and *dhps* [11]. Artesunate plus SP efficacy studies carried out in three NE states (Mizoram, Tripura and Arunachal Pradesh) in 2012 revealed treatment failure rates of >10%, necessitating a change in drug policy [12]. From 2013 onwards, artesunate plus SP was replaced by AL as the first line anti-malarial in NE states, while in the rest of the country, artesunate plus SP continues to remain as the first line of treatment [11,12]. CQ and SP drug-resistance studies carried out in NE are given in Table 4.

The first therapeutic efficacy study on ACT was carried out by Indian Council of Medical Research (ICMR) Regional Medical Research Centre, NE Region, Dibrugarh during 2004–2006 in 4 sites along the Indo-Myanmar border. This is long before ACT was introduced as a first line anti-malarial for treating *Pf* cases in NE. The study recorded 95.5% Adequate Clinical and Parasitological Response (ACPR) of ACT with mean parasite clearance time of 55:17±14:26 h. It is significant that 4.5% of the cases failed ACT (late clinical failure: 2.1%; and late parasitological failure: 2.4%) [11]. Later studies have identified the failure in ACT is due to the resistance to the partner drug, SP [11,12]. The genetic determinant of artemisinin (ART) resistance has been linked to mutations in the propeller domain of the kelch protein (*Pfkelch13* gene) [30]. Molecular screening of *Pfkelch13* by ICMR National Institute of Malaria Research (NIMR) has identified one F446I (*n* =85) and one R561H (*n* =24) validated ART resistance marker mutations in the Changlang district of Arunachal Pradesh that borders Myanmar [31,32]. In addition, few non-/synonymous mutations (K189T, A578S, and G533A) have also been reported in NE states [31,32]. Both the K189T and A578S mutations were observed in Lunglei district, Mizoram [31]. However, all the reported mutations were not associated with any decline in the clinical efficacy of ACT [31,32,33].

In vitro ring stage survival assay (RSA) carried out on 12 NE isolates obtained from Assam, Arunachal Pradesh and Tripura found that 8 out of 12 (66%) had a greater than 1% survival rate [34]. One of the NE isolates from Assam carried an A675V mutation, a candidate/associate marker, but was not associated with the slow clearance phenotype [34]. In addition, 8 of the 12 isolates had an Asn-Asn (NN) insertion between codon 142 and 143 in the *Pfkelch13* gene. Worryingly, the first report of partial ART resistance has been in the Purulia, Bankura and Kolkata regions of West Bengal [35], a state that lies in close proximity to Assam. The widespread ART in SEA necessitates the continuous monitoring of the Indo-Myanmar border to aid malaria control and prevention strategies.

Doxycycline has been recommended by NVBDCP as a short-term prophylaxis (less than 6 weeks) for selective groups from non-endemic areas such as military, para-military forces and travelers [36]. However it is rarely used, and no resistance has been observed so far.

**Figure 4:**
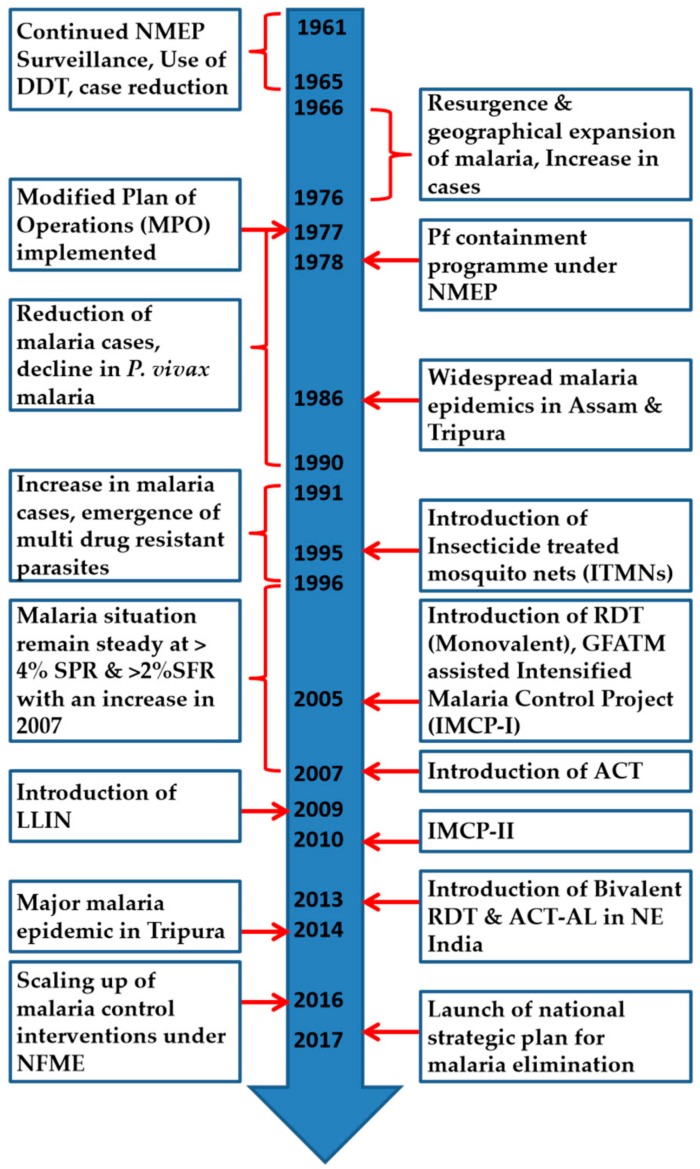
Summary of key events related to Malaria prevention and control in the NE (DDT: Dichlorodiphenyltrichloroethane, NMEP: National Malaria Eradication Program, GFATM: Global Fund to Fight AIDS, Tuberculosis and Malaria, LLIN: Long-lasting insecticide nets, ACT: Artemisinin-based combination therapy, RDT: Rapid diagnostic kit, ACT-AL: ACT Artemether-lumefantrine, NFME: National Framework for Malaria Elimination).

The summary of key events related to malaria in the NE is given in Figure 4.

**Table 4:** Drug-resistance studies in NE India.

Year	Anti-Malarial Drug	Assay Method	State/Area	Outcome	Ref No.
1973	CQ	Therapeutic efficacy	Assam	RI level of resistance in 52.5% and RII level in 22.5% of cases*	[25]
	CQ (1500 mg)	28 days *in vivo*	Garohills, Meghalaya	RI-33%, RII-9.5%, RIII-12.5%	[37]
1974	CQ	Therapeutic efficacy	Assam	RI-24%	[38]
	Quinine + pyrimethamine			No resistance observed	
1975	CQ (1500 mg)	28 days *in vivo*	Burnihat, Meghalaya	RI-78.3%, RII-4.3%	[37]
1976	CQ (1500 mg)	7 days *in vivo*	Tezu, Lohit, AP	RI-17.9%, RII-17.9%, RIII-49.2%	
1977	CQ (900 mg) + Pyrimethamine (50 mg)	7 days *in vivo*	Assam (different districts)	0–33.4% resistance	[39]
	CQ (600 mg) + Pyrimethamine (50 mg)		Assam (different districts)	0–90.0% resistance	
	CQ (600 mg) + Pyrimethamine (45 mg)		Karbi Anglong, Assam	46% resistance	
	CQ (600 mg) + Pyrimethamine (50 mg)		Nagaland	51.2% resistance	[40]
	CQ (900 mg) + Pyrimethamine (50 mg)			22% resistance	
	CQ (600 mg) + Pyrimethamine (45 mg)			47% resistance	
	CQ (1500 mg)		Bama Bazar, Garohills, Meghalaya	S/RI-82.2%, RIII-18%	[37]
	CQ (600 mg) + Pyrimethamine (50 mg)		Miao, AP	29.9% resistance	[41]
	CQ (600 mg) + Pyrimethamine (50 mg)		North Tripura	No resistance observed	
	CQ (600 mg) + Pyrimethamine (50 mg)		Aizawl, Mizoram	29% resistance	
	CQ (600 mg) + Pyrimethamine (50 mg)		Jiribam, Manipur	No resistance observed	
1978	CQ (1500 mg)	7 days *in vivo*	Karbi Anglong, Assam	66.6% resistance	[39]
1979	CQ (1500 mg)	28 days *in vivo*	Mendipathar, Garohills, Meghalaya	RI-50%	[37]
1995	CQ	3 days *in vivo* test	Assam	S/RI-85%, RI-7%, RII-3%, RIII-5%	[42]
	Alpha-Beta Arteether	28 days therapeutic efficacy	Dibrugarh, Assam	No resistance observed	[43]
1999	CQ	28 days *in vivo*	Jairampur, AP (Indo-Myanmar Border)	ETF-60.4%, LTF-22.6%	[44]
	SP	do	do	ETF-32.6%, LTF-11.6%	
	quinine	7 days *in vivo*	do	15.8% treatment failure observed	
2001	CQ	42 days prospective randomized non-blinded trial	Bokajan, Karbi Anglong, Assam	RI-53%, RII-2.2%, RIII-8.9%	[45]
	SP		Sonitpur, Assam	RI-29%, RII-3.8%, RIII-5.8%	
	Mefloquine			RI-4.4%	
	Mefloquine+artesunate			RI-8.7%, RIII-2%	
	CQ			RI-37.5%, RII-29%, RIII-29%	
	SP			RI-36.7%, RII-14.3%, RIII-6.1%	
	Mefloquine			RI-5.9%, RII-2%	
	Mefloquine+artesunate			RI-1.9%	
2002–2003	CQ	28 days *in vivo*	AP	ETF-23.8%, LCF-14.3%, LPF-10.7%	[46]
	SP		AP	ETF-14.1%, LCF-12.6%, LPF-8.1%	
2005	CQ	35 days *in vivo*	Assam	RI-13%, RII-4%, RIII-Nil	[47]
	SP			No resistance	
2007–2010	CQ	Molecular (Nested PCR)	Sonitpur, Assam	*pfcrt* K76T-72.13%, *pfmdr*1 N86Y-41.79%, K76T + N86Y-32.7%	[48]
2010	CQ	In vivo therapeutic efficacy	Tripura (Indo-Bangladesh Border)	~30% ETF, 5% LTF	[49]
2011	CQ	28 days *in vivo*	Tripura	ETF-32.5%, LCF-35%,	[50]

(CQ: Chloroquine, SP: Sulfadoxine/Pyrimethamine, *Classification of anti-malarial treatment types can be found in Box A2).

## 5. Vectors of NE India

The tropical climate provides a fertile breeding ground for varied mosquito fauna [51]. In India, 61 species of anophelines have been recorded within the subgenera *Anopheles* and *Celia*, and there is a possibility of this number increasing with identification of new species under several groups or complexes [52]. Among the 61 *Anopheles* spp., 9 have been incriminated as malaria vectors in India [53,54]. NE India is part of the “Indo-Burma hot-spot” [55], and is home to diverse fauna and flora. In the early 1990s, Malhotra and Mahanta reported 37 anophelines from NE India [56]. A later survey in early 2000s documented 45 anophelines from this region [P. Dutta, Regional Medical Research Centre, Dibrugarh personal communication]. *Anopheles dirus* and *An. minimus* complex mosquitoes are regarded as the main malaria vectors in NE India [8], and these mosquitoes inhabit distinct ecological niches.

The Minimus complex includes three formally named species: *An. minimus* (earlier species A), *An. harrisoni* (earlier species C) and *An*. *yaeyamaensis* (earlier species E). Present day taxonomy of the *An. minimus* complex has been resolved by painstaking work of several groups [57,58,59,60]. *An. minimus* was considered to be the most important malaria vector in the entire sub-Himalayan belt. Due to the large-scale application of dichlorodiphenyltrichloroethane (DDT) under the National Malaria Eradication Program (NMEP), this species was thought to have disappeared from this region. However, it re-emerged in the 1980s and was incriminated as a malaria vector in Assam [61,62], Arunachal Pradesh [63], Nagaland [64] and Mizoram [65]. In NE India, only *An. minimus* s.s. (species A) has been reported [8]. *An. minimus* is found throughout the year with higher densities between March and August, and is endophagic, endophillic and highly anthropophillic. It bites humans throughout the night, and the peak biting period is between 01:00 to 04:00AM. It breeds in slow-flowing streams with grassy banks [66], and is susceptible to DDT [67]. *An. minimus* is a highly efficient malaria vector and is responsible for focal disease outbreaks characterized by high *Pf* infections and death in forest fringe and foot hills areas of NE. Even though it is susceptible to DDT, behavioural resistance and avoidance of both DDT and long-lasting insecticide nets (LLIN) have been observed [68].

The Minimus and Fluviatilis complexes are phylogenetically [69] and morphologically closely related and are often misidentified [70]. Originally identified as *An. fluviatilis* in NE India, it is now recognized as a hyper-melanic form of *An. minimus* (species A) based on molecular data [70]. Both *An. minimus* and its hyper-melanic form were found in sympatry during winter season, and were also incriminated in malaria transmission [68].

**Figure 5:**
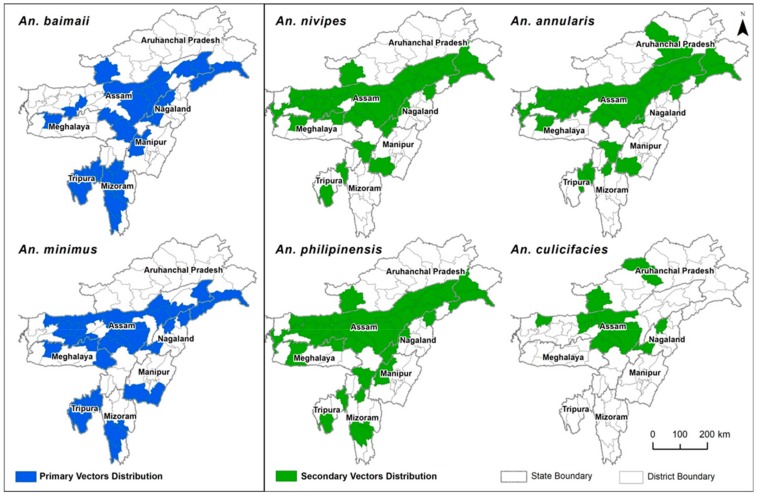
Distribution of primary and secondary malaria vectors in NE India.

*An. baimaii* is the widely prevalent species of *An. dirus* complex mosquitoes in the entire NE, along with a focal presence of *An. dirus* X (another unnamed species in the complex) in North Cachar [9]. *An. baimaii* is a forest-dwelling species found in deep forested areas and in forest borders. High anthropophilicity along with its capacity to bite equally both indoor and outdoor, and resting behavior (outside houses) makes it one of the most efficient primary malaria vectors in SEA including NE [71]. Even though the peak biting time of *An. baimaii* has been estimated to be from 22:00 to 02:00 h, in the NE it has been observed that human feeding starts from 18:00 h, and an estimated 13% of biting occurs in the 1st quarter of the night alone [71,72]. *An. baimaii* inhabits most of the hilly and forested areas of NE bordering Myanmar and Bangladesh, and most of the drug resistant cases have been recorded from these areas. Literature search shows that CQ resistant *Pf* has been commonly associated with *An. dirus* s.l. [65,73,74,75]. In NE, *An. baimaii* is susceptible to many insecticides. Due to its exophillic nature, conventional use of indoor residual insecticides is ineffective, which is also the major reason for sustained transmission of malaria in *An. baimaii*-dominated areas. Lack of an effective control strategy against *An. baimaii* is the major reason for its large population size (*Ne*), high genetic diversity and lack of population bottleneck in its evolutionary history in the NE [76]. The distribution of primary malaria vectors (*An. baimaii* and *An. minimus*) of NE is given in Figure 5. Bionomics of primary malaria vectors are given in Table 5.

## 6. Role of Secondary Vectors

Apart from these 2 primary vectors, *An. phillipinensis, An. nivipes, An. annularis, An. culicifacies* and *An. vagus* have been found to play an important and interesting role in malaria transmission, especially in the winter seasons [77]. Sporadic reports of parasite positivity in these species have been reported in the NE ever since 1941, when Anderson and Viswanathan [78] first incriminated *An. culicifacies* mosquitoes as a malaria vector in Assam. Similarly, *An. phillipinensis* has been incriminated as a malaria vector from Burnihut area of Meghalaya during 1968–1969 [79] Since then, many other anophelines have been incriminated as malaria vectors in this region (Table 6), however, their actual role in transmission of malaria is still not clear. The distribution of secondary malaria vectors are shown in Figure 5. Another important aspect of these secondary vectors is that most of them display a wide spectrum of metabolic, physiological and behavioral resistance against many insecticides [80,81,82]. Due to deforestation, shift in agricultural activities, urbanization and climate change, many natural habitats of established malaria vectors are shrinking, and their place has gradually been taken over by other invading species such as *An. culicifacies* in NE. Therefore, it is important to monitor the vectorial competency of these secondary vectors under changing climate to inform and aid the malaria control program.

**Table 5:** Bionomics of major malaria vectors in NE India.

Species	Sibling Species Present	Range	Bionomical Characteristics					Disease Transmission Relationships	
			Larval Habitat	Resting Habitat	Feeding Habit	Peak Biting Activity (hours)	Susceptibility Status to Insecticides	Density	Infectivity
*An. dirus* complex	*An. baimaii* (Dirus D) [8,9]	All NE Indian states except for Sikkim [9]	Water pits, elephant footprints, rock bed ravine, mud pools etc.	Outdoor	Highly anthropophillic, both indoor and outdoor biter [72]	21:00–03:00	Susceptible [83]	5.78# (1986–1988, Arunachal Pradesh) [84]; 0.39* (1989–1990, Assam) [66]; 5.03* (2000–2001, Assam) [85]; 1.54# (2007–2008, Tripura) [49]; 4.0–6.0* (2012, Tripura) [83]; 4.0* (2014, Tripura) [86]	0.97% (1986–1988, Arunachal Pradesh) [84]; 1.6% (Aug1995-July1996, Assam) [87]; 3.2% (June 1999 to May 2000, Assam) [88]; 0.7% sporozoite rate (2000, Assam) [85]; 1.9% (1995–2000, Assam) [71]; 11% (2014, Tripura) [86]
*An. minimus* complex	*An. minimus* (Sibling Sp. A) [8]	All NE Indian states except for Sikkim	Perennial foothill seepage water streams	Human-dwellings indoors	Highly anthropophillic	01:00-04:00	Susceptible [83]	1.86# (1986–1988, Arunachal Pradesh) [84]; 0.4* (2014, Tripura) [86]	0.40% (1986–1988, Arunachal Pradesh) [84] 0.05% (2012, Tripura) [83].

* Per person per night, #—Trap density per night.

**Table 6:** Secondary malaria vectors in NE India.

Sl. No.	Year of Incrimination	Month/Season	Place	Species	Total Positivity	Method of Incrimination	Reference
1	1941	Not Known	Assam	*An. culicifacies*	0.56%	Dissection	[78]
2	1968	August	Burnihat, Meghalaya	*An. phillipinensis*	1 no	Dissection	[79]
3	1969	July	Burnihat, Meghalaya		1 no	Dissection	
4	2001–2002	Throughout the year	Jorhat, Assam	*An. aconitus*	3.95%	CSP ELISA	[77]
				*An. annularis*	5.80%		
				*An. hyrcanus gp*	0.48%		
				*An. jeyporiensis*	6.25%		
				*An. kochi*	1.28%		
				*An. phillipinensis/nivipes*	0.94%		
				*An. tesellatus*	6.67%		
				*An. varuna*	3.23%		
				*An. vagus*	3.87%		
5	2000–2002	Throughout the year	Jairampur, AP	*An. Phillipinensis/nivipes*	1.20%	CSP ELISA	[89]
			Soraipung, DBR, Assam		2.60%		
			Titabor, Assam		1.70%		
6	2008–2009	April-October	Jorhat, Assam	*An. nivipes*	0.70%	PCR	[10]
			Dimapur, Nagaland		0.95%		
7	2012	Pre & Post monsoon	Assam	*An. culicifacies*	2.50%	CSP ELISA	[90]
			Meghalaya		3.50%		
			Manipur		1.20%		
	2013		Assam		10.90%		
			Meghalaya		6.40%		
			Manipur		1.80%		
	2014		Assam		6.60%		
			Meghalaya		3.50%		
			Manipur		9.50%		
8	2013	March-August	Orang, Assam	*An. vagus*	0.56*	CSP-ICT	[91]
			Orang, Assam	*An. annularis*	0.22*		
			Balipara, Assam	*An. vagus*	0.13*		

(* Minimum infection rate, CSP: Circumsporozoite protein, ELISA: Enzyme-linked immunosorbant assay, PCR: Polymerase chain reaction, ICT: Immunochromatographic test).

## 7. Conclusions and Future Directions

Malaria in the NE is highly complex and multi-faceted due to its unique climatic and ecological conditions and, topographic and socio-demographic heterogeneity. In the past, NE has witnessed decline and surge in malaria cases. The significant decline in malaria cases is usually associated with the introduction of new drug or intervention strategies. Also, the emergence of drug resistance, especially to *Pf,* has led to a resurgence of malaria in this region. First resistance to CQ and SP in India have been reported from NE, and the origin of these resistant parasites can be traced to SEA. With the threat of ART resistance looming in India, it is critical to step up active surveillance in NE states, especially those that border Myanmar. Strict adherence and compliance to the existing drug policy is critical in preventing and controlling drug resistance in the region.

**Figure 6:**
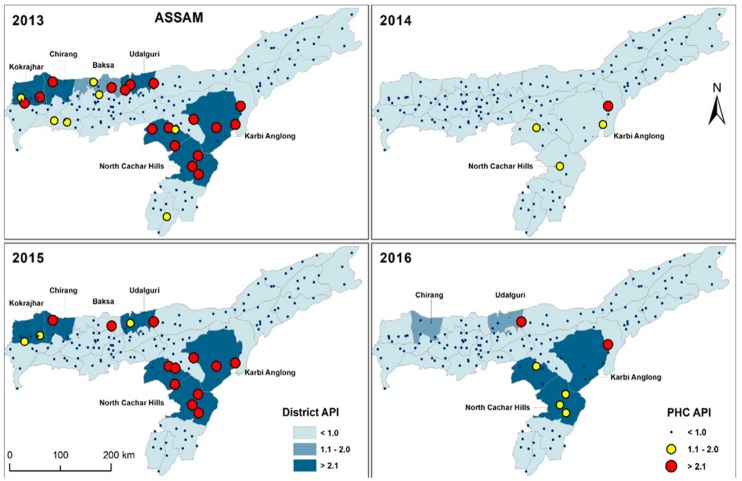
District and primary health center (PHC)-level malaria hotspot in Assam from 2013 to 2016. Malaria hotspots were calculated based on the annual parasite incidence (API) of the respective districts and PHCs.

Epidemiological trend indicates that there has been a gradual decrease in cases over the past 11 years in NE. This decline in malaria cases can be attributed to the combined effect of newer and effective drugs (Artemisinin), effective diagnostic system (use of bivalent RDTs) and implementation of effective mosquito control strategies such as LLINs. Even though the cases have decreased, Assam, Arunachal Pradesh, Tripura and Mizoram continue to be malaria-endemic states. As part of the control strategy, high transmission zones or malaria hot-spots in these regions need to be mapped, and have to be continuously monitored. Malaria hotspots from 2013 to 2016 based on annual parasite incidence (API) at district and PHC level in Assam mapped by Geographic Information system (GIS) are shown in Figure 6. Malaria intervention strategies have to be focused on these mapped high-transmission settings. Similar hot spot mapping have to be carried out in other NE states for effective malaria control and elimination in this region.

The true burden of asymptomatic malaria carriers have to be carefully ascertained in these malaria endemic states. Asymptomatic carriers are individuals who carry the malaria parasites without manifesting any clinical symptoms. However, they remain infectious to mosquitoes and continue to spread the disease [92], and they are potential threat to malaria elimination efforts [93]. It is estimated that 20%–70% of the infections cannot be detected by conventional microscopy and RDTs [94]. Active surveillance using molecular tools and treatment of asymptomatic carriers should be given high priority as part of malaria elimination efforts in this region.

Even though *Pf* is the major cause of malaria in the malaria-endemic states of Tripura, Meghalaya, Assam and Mizoram, a significant number of cases of *Pv* are reported every year. Furthermore, in the states of Arunachal and Nagaland, *Pv* is the leading cause of malaria. In these states, *Pv* cases were recorded throughout the year with a peak during the rainy season and children less than 5 years old were more affected [22]. It is important to understand the relapse pattern and transmission dynamics of *Pv* in different eco-epidemiological settings for effective control and elimination of *Pv* in the NE. Also, the status of CQR in *Pv* has to be continuously monitored in all NE states.

Most of the vector biology studies in the NE have focused around *An. baimaii* and *An. minimus*, the major malaria vectors. Climate change, deforestation, urbanization and migration have had a great impact on the current habitat of these established malaria vectors. The natural habitat of the primary vectors are shrinking, and in parallel, this has created habitats for new and emerging malaria vectors. Several studies have reported the emergence of secondary malaria vectors in the NE. The vectorial capacity of other *Anophelines* in different ecological and epidemiological settings of the NE have to be studied to ascertain their role in malaria epidemiology in this region. *An. subpictus* incriminated as a major malaria vector in Western India [95] has been identified in mosquito epidemiology studies in Mizoram [96]. Substantial focus has to be given to understanding the emergence of new vectors and vector parasite relationships for formulating effective vector-control strategies in this region.

Overall, malaria control and elimination in the NE will be an important step in India’s efforts to eradicate malaria in the next decade.

## Figures and Tables

**Table 1:** Salient features of malaria in North-East (NE) India.

NE States	Assam, Arunachal Pradesh, Mizoram, Tripura, Meghalaya, Nagaland and Manipur
Geographical Area	255,128 Km^2^ (consists of hills, valleys and plains)
National proportion to land cover	8%
Total Population	45161611 (2011 census) with an average density of 159 persons/km^2^
Climate	Tropical monsoon humid climate
Contribution to nation’s malaria cases	15.2% (in 2018)
Major *Plasmodium* spp.	*P. falciparum* and *P. vivax*
Primary malaria vectors	*An. baimaii* and *An. minimus*
Secondary malaria vectors	*An. annularis, An. phillipinensis/nivipes*, *An. culicifacies etc.*
Treatment for malaria	*P. falciparum*—Age-specific artemisinin combination therapy (Artemether-lumefantrine) with primaquine @ 0.75 mg/kg body weight on day 2 *P. vivax*—chloroquine (25 mg/kg body weight divided over three days) and primaquine (0.25 mg/kg body weight daily for 14 days.) For mixed infections—Age-specific artemisinin combination therapy (Artemether-lumefantrine) for 3 days + primaquine 0.25 mg per kg body weight daily for 14 days.

**Table 2:** Distribution of malaria cases in 7 NE states from 2008 to 2018 (Source: National Vector Borne Disease Control Program (NVBDCP), India).

States	Assam			Arunachal Pradesh			Meghalaya			Mizoram			Nagaland			Manipur			Tripura		
Year	Number of Malaria Cases	*Plasmodium falciparum*	*Plasmodium vivax*	Number of Malaria Cases	*Plasmodium falciparum*	*Plasmodium vivax*	Number of Malaria Cases	*Plasmodium falciparum*	*Plasmodium vivax*	Number of Malaria Cases	*Plasmodium falciparum*	*Plasmodium vivax*	Number of Malaria Cases	*Plasmodium falciparum*	*Plasmodium vivax*	Number of Malaria Cases	*Plasmodium falciparum*	*Plasmodium vivax*	Number of Malaria Cases	*Plasmodium falciparum*	*Plasmodium vivax*
2008	83,939	58,124	25,815	29,146	8219	20,927	39,616	36,301	3315	7361	6172	1189	5078	835	4243	708	356	352	25,894	23,588	2306
2009	91,413	66,557	24,856	22,066	6602	15,464	76,759	74,251	2508	9399	7387	2012	8489	2893	5596	1069	620	449	24,430	22,952	1478
2010	68,353	48,330	20,023	17,944	5412	12,532	41,642	39,374	2268	15,594	14,664	930	4959	1877	3082	947	487	460	23,939	21,254	2685
2011	47,397	34,707	12,690	13,950	4856	9094	25,143	24,018	1125	8861	8373	488	3363	950	2413	714	314	400	14,417	13,812	605
2012	29,999	20,579	9420	8368	2789	5579	20,834	19,805	1029	9883	9437	446	2891	821	2070	255	83	172	11,565	10,915	650
2013	19,542	14,969	4573	6398	2181	4217	24,727	22,885	1842	11,747	10,340	1407	2285	519	1766	120	42	78	7396	6998	398
2014	14,540	11,210	3330	6082	2338	3744	39,168	37,149	2019	23,145	21,083	2062	1936	647	1289	145	72	73	51,240	49,653	1587
2015	15,557	11,675	3882	5088	1714	3374	48,603	43,828	4775	28,593	24,602	3991	1527	532	995	216	119	97	32,525	30,074	2451
2016	7826	5686	2140	3128	895	2233	35,147	31,867	3280	7583	5907	1676	828	316	512	122	58	64	10,546	9545	1001
2017	5281	3494	1787	1546	488	1058	16,454	14,418	2036	5715	4974	741	394	188	206	80	22	58	7051	6571	480
2018	3816	2859	957	625	154	471	6394	6065	329	4296	3937	359	113	24	89	12	3	9	13,079	12,600	479

**Table 3:** Malaria cases in proportion to population.

State	*Pf* Cases	*Pv* Cases	Total Cases (2008–2018)	Density (2011)	Population (2011)	Average Cases/Year (2008–2018)	Average Cases to Population	Total Cases to Population
Arunachal Pradesh	35,648	78,693	114,341	17	1,382,611	10,394.63	0.75%	8.3%
Assam	278,190	109,473	387,663	397	31,169,272	35,242.09	0.11%	1.2%
Manipur	2176	2212	4388	122	2,721,756	398.90	0.01%	0.2%
Meghalaya	349,961	24,526	374,487	132	2,964,007	34,044.27	1.15%	12.6%
Mizoram	116,876	15,301	132,177	52	1,091,014	12,016.09	1.10%	12.1%
Nagaland	9602	22,261	31,863	119	1,980,602	2896.63	0.15%	1.6%
Tripura	207,962	14,120	222,082	350	3,671,032	20,189.27	0.55%	6.0%

## Appendix A

Box A1 Definition of epidemiological parameters used in malaria. **Annual parasitic incidence (API)**: Number of new infections per year per 1000 population (Total no. of positive slides for parasite in a year × 1000/Total population. API - (ABER*SPR)/10 **Incidence:** Number of newly diagnosed malaria cases during a defined period in a specified population **Malaria mortality rate:** Number of deaths from malaria per unit of population during a defined period **Prevalence:** Proportion of a specified population with malaria infection at one time **Slide positivity rate (SPR)**: Proportion of blood smears found to be positive for *Plasmodium* among all blood smears examined (Total positive × 100/Total slides examined) **Slide *falciparum* rate (SFR)**: Proportion of blood smears found to be positive for *Pf* among all blood smears examined (Total positive *Pf* 100/Slides examined)

Box A2 Classification of responses to anti-malarial treatment [97]. **RI:** Recrudescence of the infection between 7 and 28 days after completion of treatment following initial resolution of symptoms and parasite clearance. **RII:** Reduction of parasitaemia by >75% at 48 h but failure to clear parasites within 7 days. **RIII:** Parasitaemia does not fall by >75% within 48 h
**Early treatment failure (ETF):**
    Danger signs or severe malaria on day 1, 2 or 3, in the presence of parasitaemia;    Parasitaemia on day 2 higher than on day 0, irrespective of axillary temperature;    Parasitaemia on day 3 with axillary temperature ≥37.5 °C; and    Parasitaemia on day 3 ≥25% of count on day 0.
**Late clinical failure (LCF):**
    Danger signs or severe malaria in the presence of parasitaemia on any day between day 4 and day 28 (day 42) in patients who did not previously meet any of the criteria of early treatment failure; and    Presence of parasitaemia on any day between day 4 and day 28 (day 42) with axillary temperature ≥37.5 °C in patients who did not previously meet any of the criteria of early treatment failure.
**Late parasitological failure (LPF):**
    Presence of parasitaemia on any day between day 7 and day 28 (day 42) with axillary temperature <37.5 °C in patients who did not previously meet any of the criteria of early treatment failure or late clinical failure.
**Adequate clinical and parasitological response (ACPR):**
    Absence of parasitaemia on day 28 (day 42), irrespective of axillary temperature, in patients who did not previously meet any of the criteria of early treatment failure, late clinical failure or late parasitological failure.

## References

[1] WHO (2018). World malaria report.

[2] https://www.nvbdcp.gov.in/WriteReadData/l892s/95838975781568980798.pdf.

[3] Wangdi K., Gatton M.L., Kelly G.C., Banwell C., Dev V., Clements A.C.A. (2016). Malaria elimination in India and regional implications. Lancet Infect. Dis..

[4] https://www.who.int/malaria/publications/country-profiles/profile_ind_en.pdf?ua=1.

[5] Dev V., Bhattacharyya P.C., Talukdar R. (2003). Transmission of malaria and its control in the northeastern region of India. J. Assoc. Physicians India.

[6] Mohapatra P.K., Prakash A., Bhattacharyya D.R., Goswami B.K., Ahmed A., Sarmah B., Mahanta J. (2008). Detection & molecular confirmation of a focus of *Plasmodium malariae* in Arunachal Pradesh, India. Indian J. Med. Res..

[7] Prakash A., Mohapatra P., Bhattacharyya D., Goswami B.K., Mahanta J. (2003). *Plasmodium ovale*: First case report from Assam, India. Curr. Sci..

[8] Prakash A., Walton C., Bhattacharyya D.R., Loughlin S.O., Mohapatra P.K., Mahanta J. (2006). Molecular characterization and species identification of the Anopheles dirus and *An. minimus* complexes in north-east India using r-DNA ITS-2. Acta Trop..

[9] Prakash A., Sarma D.K., Bhattacharyya D.R., Mohapatra P.K., Bhattacharjee K., Das K., Mahanta J. (2010). Spatial distribution and r-DNA second internal transcribed spacer characterization of *Anopheles dirus* (Diptera: Culicidae) complex species in north-east India. Acta Trop..

[10] Bhattacharyya D.R., Prakash A., Sarma N.P., Mohapatra P.K., Singh S., Sarma D.K., Kalita M.C., Mahanta J. (2010). Molecular evidence for the involvement of *Anopheles nivipes* (Diptera: Culicidae) in the transmission of Plasmodium falciparum in north-eastern India. Ann. Trop. Med. Parasitol..

[11] Mohapatra P.K., Sarma D.K., Prakash A., Bora K., Ahmed M.A., Sarma B., Goswami B.K., Bhattacharyya D.R., Mahanta J. (2014). Molecular evidence of increased resistance to anti-folate drugs in *Plasmodium falciparum* in North-East India: A signal for potential failure of artemisinin plus sulphadoxine-pyrimethamine combination therapy. PLoS ONE.

[12] Mishra N., Kaitholia K., Srivastava B., Shah N.K., Narayan J.P., Dev V., Phookan S., Anvikar A.R., Rana R., Bharti R.S. (2014). Declining efficacy of artesunate plus sulphadoxine-pyrimethamine in northeastern India. Malar. J..

[13] WHO-SEARO (2016). India Launches the National Framework to Eliminate Malaria.

[14] Ghosh S.K., Rahi M. (2019). Malaria elimination in India-The way forward. J. Vector Borne Dis..

[15] WHO (2015). World Malaria Report.

[16] APMEN (2015). Elimination 2030. Asia Pacific malaria elimination network. https://www.apmen.org/web/1/elimination/.

[17] Dikshit K.R., Dikshit J.K., Dikshit K.R., Dikshit J.K. (2014). Relief Features of North-East India. North-East India: Land, People and Economy.

[18] Dikshit K.R., Dikshit J.K. (2014). Weather and Climate of North-East India. North-East India: Land, People and Economy.

[19] Dikshit K.R., Dikshit J.K. (2014). Natural Vegetation: Forests and Grasslands of North-East India. North-East India: Land, People and Economy.

[20] Dikshit K.R., Dikshit J.K. (2014). Population of the North-Eastern States of India. North-East India: Land, People and Economy.

[21] Mohapatra P.K., Prakash A., Bhattacharyya D.R., Mahanta J. (1998). Malaria situation in north-eastern region of India. Icmr Bull..

[22] Sharma V.P., Dev V., Phookan S. (2015). Neglected *Plasmodium vivax* malaria in northeastern States of India. Indian J. Med. Res..

[23] Fairhurst R.M., Dondorp A.M. (2016). Artemisinin-Resistant *Plasmodium falciparum* Malaria. Microbiol. Spectr..

[24] Payne D. (1987). Spread of chloroquine resistance in *Plasmodium falciparum*. Parasitol. Today.

[25] Sehgal P.N., Sharma M.I.D., Sharma S.L., Gogai S. (1973). Resistance to chloroquine in falciparum malaria in Assam State, India. J. Commun. Dis..

[26] Shah N.K., Dhillon G.P., Dash A.P., Arora U., Meshnick S.R., Valecha N. (2011). Antimalarial drug resistance of *Plasmodium falciparum* in India: Changes over time and space. Lancet Infect. Dis..

[27] https://nvbdcp.gov.in/Doc/drug-policy-2010.pdf.

[28] Mishra N., Singh J.P., Srivastava B., Arora U., Shah N.K., Ghosh S.K., Bhatt R.M., Sharma S.K., Das M.K., Kumar A. (2012). Monitoring antimalarial drug resistance in India via sentinel sites: Outcomes and risk factors for treatment failure, 2009–2010. Bull. World Health Organ..

[29] Das S.B.B., Roy J.R., Guha A.K., Rastogi A.C. (1981). Pyrimethamine in combination with sulfadoxine or sulfalene in *P. falciparum* infected cases in India. Indian J. Malariol..

[30] Ariey F., Witkowski B., Amaratunga C., Beghain J., Langlois A.C., Khim N., Kim S., Duru V., Bouchier C., Ma L. (2014). A molecular marker of artemisinin-resistant *Plasmodium falciparum* malaria. Nature.

[31] Mishra N., Bharti R.S., Mallick P., Singh O.P., Srivastava B., Rana R., Phookan S., Gupta H.P., Ringwald P., Valecha N. (2016). Emerging polymorphisms in falciparum Kelch 13 gene in Northeastern region of India. Malar. J..

[32] Mishra N., Prajapati S.K., Kaitholia K., Bharti R.S., Srivastava B., Phookan S., Anvikar A.R., Dev V., Sonal G.S., Dhariwal A.C. (2015). Surveillance of artemisinin resistance in *Plasmodium falciparum* in India using the kelch13 molecular marker. Antimicrob. Agents Chemother..

[33] Mishra S., Bharti P.K., Shukla M.M., Ali N.A., Kashyotia S.S., Kumar A., Dhariwal A.C., Singh N. (2017). Clinical and molecular monitoring of Plasmodium falciparum resistance to antimalarial drug (artesunate+sulphadoxine-pyrimethamine) in two highly malarious district of Madhya Pradesh, Central India from 2012–2014. Pathog. Glob. Health.

[34] Chakrabarti R., White J., Babar P.H., Kumar S., Mudeppa D.G., Mascarenhas A., Pereira L., Dash R., Maki J.N., Sharma A. (2019). Decreased in vitro artemisinin sensitivity of *Plasmodium falciparum* across India. Antimicrob. Agents Chemother..

[35] Das S., Manna S., Saha B., Hati A.K., Roy S. (2019). Novel pfkelch13 Gene Polymorphism Associates With Artemisinin Resistance in Eastern India. Clin. Infect. Dis. Off. Publ. Infect. Dis. Soc. Am..

[36] Anvikar A. (2011). Guidelines for Diagnosis and Treatment of Malaria in India.

[37] Chakravarty S.C., Dwivedi S.R., Das S., Pukan D., Roy R.G., Pattanayak S. (1979). Response of *Plasmodium falciparum* to chloroquine in the Meghalaya state. Indian J. Med. Res..

[38] Sehgal P.N., Sharma S.S. (1974). Efficacy of quinine with pyrimethamine against chloroquine resistant *P. falciparum* malaria in Assam State India. J. Commun. Dis..

[39] Pattanayak S., Roy R.G., Phukan D., Barkakuty B.N. (1979). Chloroquine resistance in *P. falciparum* in Assam State. Indian J. Med. Res..

[40] Das S., Roy R.G., Pattanayak S. (1979). A note on chloroquine resistance tests on *P. falciparum* in Nagaland. Indian J. Med. Res..

[41] Pattanayak S., Roy R.G., Sen N. (1979). Response of Chloroquine with or without pyrimethamine in *Plasmodium falciparum* in West Bengal, Tripura, Mizoram, Manipur and Arunachal Pradesh. Indian J. Med. Res..

[42] Gogoi S.C., Dev V., Choudhury B., Phookan S. (1995). Susceptibility of *Plasmodium falciparum* to chloroquine in tea garden tribes of Assam, India. Southeast Asian J. Trop. Med. Public Health.

[43] Mohapatra P.K., Khan A.M., Prakash A., Mahanta J., Srivastava V.K. (1996). Effect of arteether alpha/beta on uncomplicated falciparum malaria cases in Upper Assam. Indian J. Med. Res..

[44] Mohapatra P.K., Namchoom N.S., Prakash A., Bhattacharya D.R., Goswami B.K., Mahanta J. (2003). Therapeutic efficacy of anti-malarials in *Plasmodium falciparum* malaria in an Indo-Myanmar border area of Arunachal Pradesh. Indian J. Med. Res..

[45] Campbell P., Baruah S., Narain K., Rogers C.C. (2006). A randomized trial comparing the efficacy of four treatment regimens for uncomplicated falciparum malaria in Assam state, India. Trans. R. Soc. Trop. Med. Hyg..

[46] Mohapatra P.K., Prakash A., Taison K., Negmu K., Gohain A.C., Namchoom N.S., Wange D., Bhattacharyya D.R., Goswami B.K., Borgohain B.K. (2005). Evaluation of chloroquine (CQ) and sulphadoxine/pyrimethamine (SP) therapy in uncomplicated falciparum malaria in Indo-Myanmar border areas. Trop. Med. Int. Health.

[47] Baruah I., Talukdar P.K., Das S.C. (2005). The drug sensitivities of *Plasmodium falciparum* in the Sonitpur district, Assam, India. Southeast Asian J. Trop. Med. Public Health.

[48] Goswami D., Dhiman S., Rabha B., Kumar D., Baruah I., Veer V., Bhola R., Sharma D. (2014). High prevalence of pfcrt K76T and mdr1 N86Y mutations in Sonitpur district of Assam, India. J. Parasit. Dis. Off. Organ Indian Soc. Parasitol..

[49] Dhiman S., Goswami D., Rabha B., Gopalakrishnan R., Baruah I., Singh L. (2010). Malaria epidemiology along Indo-Bangladesh border in Tripura State, India. Southeast Asian J. Trop. Med. Public Health.

[50] Majumdar T.D.N., Roy D.B. (2011). Assessment of therapeutic efficacy of chloroquine in uncomplicated Plasmodium falciparum malaria in a rural area of Tripura. J. Pharm. Biomed. Sci..

[51] Nagpal B.N., Sharma V.P. (1995). Indian anophelines.

[52] Bhattacharyya D., Rajavel A., Natarajan R., Mohapatra P., Jambulingam P., Mahanta J., Prakash A. (2014). Faunal richness and the checklist of Indian mosquitoes (Diptera: Culicidae). Check List.

[53] Lal S., Sonal G.S., Phukar P.K. (2000). Status of malaria in India. J. Indian Acad. Clin. Med..

[54] Sharma V.P. (1999). Current scenario of malaria in India. Parassitologia.

[55] Myers N., Mittermeier R.A., Mittermeier C.G., da Fonseca G.A., Kent J. (2000). Biodiversity hotspots for conservation priorities. Nature.

[56] Malhotra P.R., Mahanta H.C. (1994). Check-List of mosquitoes of Northeast India (Diptera: Culicidae). Orient. Insects.

[57] Harbach R.E., Parkin E., Chen B., Butlin R.K. (2006). Anopheles (Cellia) minimus Theobald (Diptera: Culicidae): Neotype designation, characterization, and systematics. Proc. Entomol. Soc. Wash..

[58] Harbach R.E., Garros C., Manh N.D., Manguin S. (2007). Formal taxonomy of species C of the Anopheles minimus sibling species complex (Diptera: Culicidae). Zootaxa.

[59] Chen B., Harbach R.E., Butlin R.K. (2002). Molecular and morphological studies on the Anopheles minimus group of mosquitoes in southern China: Taxonomic review, distribution and malaria vector status. Med. Vet. Entomol..

[60] Somboon P., Rory A., Tsuda Y., Takagi M., Harbach R. (2010). Systematics of Anopheles (Cellia) yaeyamaensis sp. n. alias species E of the *An. minimus* complex in southeastern Asia (Diptera: Culicidae). Zootaxa.

[61] Kareem M.A., Singh Y.K., Bhatnagar V.N., Krishnamurthy B.S., Das M., Sharma G.K. (1985). A preliminary report on some entomological observations in malaria endemic areas of Kamrup district, Assam. J. Commun. Dis..

[62] Prakash A., Mohapatra P.K., Srivastava V.K. (1996). Vector incrimination in Tamulpur primary health centre, district Nalbari, lower Assam during malaria outbreak 1995. Indian J. Med. Res..

[63] Dutta P., Baruah B.D. (1987). Incrimination of Anopheles minimus Theobald as a vector of malaria in Arunachal Pradesh. Indian J. Malariol..

[64] Bhatnagar V.N., Dwivedi S.R., Mishra B.G., Das M. (1982). Detection and incrimination of Anopheles minimus Theobald 1901 as malaria vector in the foothill areas of Nagaland, India. Indian J. Malariol..

[65] Das S.C., Baruah I. (1985). Incrimination of Anopheles minimus Theobald and Anopheles balabacensis balabacensis Baisas (A. dirus) as malaria vectors in Mizoram. Indian J. Malariol..

[66] Dev V. (1996). Anopheles minimus: Its bionomics and role in the transmission of malaria in Assam, India. Bull. World Health Organ..

[67] Prakash A., Bhattacharya D.R., Mohapatra P.K., Mahanta J. (1998). Insecticide susceptibility of Anopheles dirus in Assam. J. Commun. Dis..

[68] Dev V., Manguin S. (2016). Biology, distribution and control of Anopheles (Cellia) minimus in the context of malaria transmission in northeastern India. Parasites Vectors.

[69] Garros C., Harbach R.E., Manguin S. (2005). Systematics and biogeographical implications of the phylogenetic relationships between members of the funestus and minimus groups of Anopheles (Diptera: Culicidae). J. Med. Entomol..

[70] Singh O.P., Nanda N., Dev V., Bali P., Sohail M., Mehrunnisa A., Adak T., Dash A.P. (2010). Molecular evidence of misidentification of Anopheles minimus as Anopheles fluviatilis in Assam (India). Acta Trop..

[71] Prakash A., Bhattacharyya D.R., Mohapatra P.K., Mahanta J. (2005). Malaria transmission risk by the mosquito *Anopheles baimaii* (formerly known as *An. dirus* species D) at different hours of the night in North-east India. Med. Vet. Entomol..

[72] Dutta P., Bhattacharyya D.R., Khan S.A., Sharma C.K., Mahanta J. (1996). Feeding patterns of Anopheles dirus, the major vector of forest malaria in north east India. Southeast Asian J. Trop. Med. Public Health.

[73] Dutta P., Bhattacharyya D.R., Sharma C.K., Dutta L.P. (1989). The importance of *Anopheles dirus* (*A. balabacensis*) as a vector of malaria in northeast India. Indian J. Malariol..

[74] Gould D.J., Cadigan F.C., Ward R.A. (1966). Falciparum malaria: Transmission to the gibbon by *Anopheles balabacensis*. Science.

[75] Yang T.H. (1983). A review of literature on Anopheles balabacensis balabacensis.

[76] Sarma D.K., Prakash A., O’Loughlin S.M., Bhattacharyya D.R., Mohapatra P.K., Bhattacharjee K., Das K., Singh S., Sarma N.P., Ahmed G.U. (2012). Genetic population structure of the malaria vector *Anopheles baimaii* in north-east India using mitochondrial DNA. Malar. J..

[77] Prakash A., Bhattacharyya D.R., Mohapatra P.K., Mahanta J. (2004). Role of the prevalent Anopheles species in the transmission of *Plasmodium falciparum* and *P. vivax* in Assam state, north-eastern India. Ann. Trop. Med. Parasitol..

[78] Anderson L.A.P., Viswanathan D.K. (1943). The Assam Medical Research Society, Shillong: A resume on its activities during 1931–1941. Calcatta:ThackerSpink Co..

[79] Rajagopal R. (1976). Studies on persistent transmission of malaria in Burnihat, Meghalaya. J. Commun. Dis..

[80] Dev V., Sharma V., Manguin S. (2013). The Dominant Mosquito Vectors of Human Malaria in India, Anopheles mosquitoes-New insights into malaria vectors, Sylvie Manguin. Anopheles Mosquitoes, New Insights Into Malaria Vectors.

[81] Dhiman S., Rabha B., Goswami D., Das N.G., Baruah I., Bhola R.K., Veer V. (2014). Insecticide resistance and human blood meal preference of Anopheles annularis in Asom-Meghalaya border area, northeast India. J. Vector Borne Dis..

[82] Raghavendra K., Velamuri P.S., Verma V., Elamathi N., Barik T.K., Bhatt R.M., Dash A.P. (2017). Temporo-spatial distribution of insecticide-resistance in Indian malaria vectors in the last quarter-century: Need for regular resistance monitoring and management. J. Vector Borne Dis..

[83] Dev V., Adak T., Singh O.P., Nanda N., Baidya B.K. (2015). Malaria transmission in Tripura: Disease distribution & determinants. Indian J. Med. Res..

[84] Dutta P., Bhattacharyya D.R., Sharma C.K., Dutta L.P. (1992). Anopheline fauna of parts of Tirap district, Arunachal Pradesh with reference to malaria transmission. Indian J. Med. Res..

[85] Prakash A., Bhattacharyya D.R., Mohapatra P.K., Barua U., Phukan A., Mahanta J. (2003). Malaria control in a forest camp in an oil exploration area of Upper Assam. Natl. Med. J. India.

[86] Sarmah N., Bhowmik I., Sarma D., Sharma C., Medhi G., Mohapatra P., Mahanta J., Bhattacharyya D. (2019). Role of Anopheles baimaii: Potential vector of epidemic outbreak in Tripura, North-east India. J. Glob. Health Rep..

[87] Prakash A., Bhattacharyya D.R., Mohapatra P.K., Mahanta J. (1997). Seasonal prevalence of Anopheles dirus and malaria transmission in a forest fringed village of Assam, India. Indian J. Malariol..

[88] Prakash A., Bhattacharyya D.R., Mohapatra P.K., Mahanta J. (2001). Estimation of vectorial capacity of *Anopheles dirus* (Diptera: Culicidae) in a forest-fringed village of Assam (India). Vector Borne Zoonotic Dis..

[89] Prakash A., Bhattacharyya D.R., Mohapatra P.K., Mahanta J. (2005). Potential of Anopheles philippinensis-nivipes complex mosquitoes as malaria vector in north-east India. J. Environ. Biol..

[90] Akhtar N., Nagpal B.N., Kapoor N., Srivastava A., Valecha N. (2016). Role of *An. culicifacies* as a vector of malaria in changing ecological scenario of Northeastern states of India. J. Vector Borne Dis..

[91] Dhiman S., Yadav K., Rabha B., Goswami D., Hazarika S., Tyagi V. (2016). Evaluation of Insecticides Susceptibility and Malaria Vector Potential of *Anopheles annularis* s.l. and Anopheles vagus in Assam, India. PLoS ONE.

[92] Lindblade K.A., Steinhardt L., Samuels A., Kachur S.P., Slutsker L. (2013). The silent threat: *Asymptomatic parasitemia* and malaria transmission. Expert Rev. Anti-Infect. Ther..

[93] Sattabongkot J., Suansomjit C., Nguitragool W., Sirichaisinthop J., Warit S., Tiensuwan M., Buates S. (2018). Prevalence of asymptomatic Plasmodium infections with sub-microscopic parasite densities in the northwestern border of Thailand: A potential threat to malaria elimination. Malar. J..

[94] Poirot E., Skarbinski J., Sinclair D., Kachur S.P., Slutsker L., Hwang J. (2013). Mass drug administration for malaria. Cochrane Database Syst. Rev..

[95] Kumar A., Hosmani R., Jadhav S., de Sousa T., Mohanty A., Naik M., Shettigar A., Kale S., Valecha N., Chery L. (2016). Anopheles subpictus carry human malaria parasites in an urban area of Western India and may facilitate perennial malaria transmission. Malar. J..

[96] Zomuanpuii R., Ringngheti L., Brindha S., Gurusubramanian G., Senthil Kumar N. (2013). ITS2 characterization and Anopheles species identification of the subgenus Cellia. Acta Trop..

[97] Rieckmann K.H. (1990). Monitoring the response of malaria infections to treatment. Bull. World Health Organ..

